# Rapid health technology assessment of six non-ergot dopamine-receptor agonists for the treatment of early Parkinson’s disease

**DOI:** 10.3389/fphar.2025.1648833

**Published:** 2025-12-17

**Authors:** Jieqiong Lv, Hongbo Fu, Yanqiong Zhou, Wenwei Li, Jiaxin Zhang, Zhihua Zhou, Sha Lai

**Affiliations:** 1 Department of Pharmacy, The Second Affiliated Hospital of Shantou University Medical College, Shantou, China; 2 Key Specialty of Clinical Pharmacy, The First Affiliated Hospital of Guangdong Pharmaceutical University, Guangzhou, China; 3 Department of Neurology, Neurological Research Institute of Integrated Traditional Chinese and Western Medicine, The First Affiliated Hospital of Guangdong Pharmaceutical University, Guangzhou, China

**Keywords:** drug selection, non-ergot dopamine-receptor agonists, Parkinson disease, piribedil, pramipexole extended-release, rapid health technology assessment

## Abstract

**Purpose:**

Six non-ergot dopamine -receptor agonists (NEDAs), including pramipexole extended-release (ER), pramipexole immediate-release (IR), rotigotine patch, piribedil, ropinirole prolonged-release (PR), and ropinirole IR, are frequently used to treat early Parkinson’s disease (PD). However, there is a paucity of comparative information about them. For making rational drug selections, six NEDAs were evaluated using a rapid health technology assessment method.

**Methods:**

According to the rapid guidelines for drug evaluation and selection in Chinese medical institutions (the Second Edition), the pharmacological properties, efficacy, safety, economy, and other attributes of six NEDAs were scored on a percentage system.

**Results:**

The composite scores of the six NEDAs are presented as follows: 74.99 points for pramipexole ER, 74.90 points for piribedil, 72.40 points for pramipexole IR, 70.78 points for rotigotine patch, 69.06 points for ropinirole PR and 68.41 points for ropinirole IR.

**Conclusion:**

Based on our comprehensive multi-criteria assessment, pramipexole ER, pramipexole IR, rotigotine patch, and piribedil are strongly recommended for inclusion in the formularies of medical institutions as therapeutic options for patients with early PD.

## 1 Introduction

Parkinson’s disease (PD) is a common neurodegenerative disease, with its incidence trailing only that of Alzheimer’s disease (Chen et al., 2023). According to a global burden of disease study (Dorsey and Bloem, 2018), the global number of patients with PD will increase from 7 million in 2015 to 13 million in 2040, indicating that PD may become more prevalent. Meanwhile, the number of PD patients in China is expected to increase to 5 million by 2030, accounting for almost half of the global PD population (Li et al., 2019). As the disease progresses, the worsening of motor and non-motor symptoms not only damages the patient’s daily activities but also places a heavy burden on society (Taximaima et al., 2021).

As first-line drugs for early PD, Non-ergot dopamine receptor agonists (NEDAs) have been recommended by authoritative guidelines (Su and Chen, 2021; Chen, 2020; Fox et al., 2018; Rascol et al., 2000; Chen, 2016; Poewe et al., 2011; Grimes et al., 2019). With the development of novel long-acting formulations of NEDAs, such as once-daily oral extended-release (ER) formulations [pramipexole ER and ropinirole prolonged-release (PR)] and rotigotine patch, these novel NEDAs have improved patient compliance and provide more stable 24-h blood drug concentrations (Ceravolo et al., 2016; Jenner et al., 2009). Multiple NEDAs offer more options for PD patients, yet they also create significant challenges in the drug selection process for physicians, leading to increased economic and social burdens for patients. However, a critical gap persists in the existing evidence. While numerous randomized controlled trials (RCTs) have assessed individual NEDAs, the literature remains fragmented and predominantly focuses on isolated clinical outcomes. There is a conspicuous lack of studies that provide a comprehensive evaluation and direct comparison of the overall clinical value of these six drugs. This fragmentation hinders the ability of healthcare decision-makers to identify which NEDAs offer the most balanced profile.

Health technology assessment (HTA) is a comprehensive and systematic method that evaluates the effectiveness, safety, economic, and social aspects of health technology, and uses theories from multiple disciplines, such as evidence-based medicine and health economics (Angelis et al., 2023). While traditional HTA delivers in-depth analysis, its resource-intensive and time-consuming nature often limits its applicability in rapidly evolving policy environments (Yun et al., 2023). In contrast, rapid HTA is an emerging method, adapted to national conditions and policies, which is characterized by its rapidity, comprehensiveness, and scientific rigour. Unlike traditional HTA, rapid HTA focuses on evaluating specific, pressing questions to provide the best available evidence within a constrained time frame (Hamel et al., 2021). Methodologically, it employs systematic yet pragmatic literature reviews, incorporates explicit time filters, and adapts continuously to new technologies and policy changes. This approach enables healthcare decision-makers and medical professionals to make informed clinical and policy decisions during critical moments, without compromising the time necessary for evidence synthesis and reporting (Wang et al., 2025; Chen et al., 2018).

To address this gap, this study employs the rapid HTA framework for the first time to evaluate the comprehensive clinical value of six NEDAs in early PD through a multidimensional evaluation system. The aim is to provide a scientific basis for drug selection and decision-making within medical institutions, as well as offer a transferable evaluation method for medical decision-making in other regions.

The specific objectives are to: (1) systematically evaluate and compare six NEDAs across multiple predefined dimensions using a standardized multi-criteria decision analysis (MCDA) framework; (2) determine whether this methodology generates distinct, quantifiable scores and rankings that reflect meaningful differences in the drugs’ overall clinical value; and (3) provide an evidence-based foundation to support clinical and policy decision-making in the management of early PD.

## 2 Drugs and methods

### 2.1 Drugs

At present, seven types of NEDAs are available for clinical application: pramipexole ER, pramipexole immediate-release (IR), rotigotine patch, piribedil, ropinirole PR, ropinirole IR, and apomorphine subcutaneous injection. However, apomorphine subcutaneous injection is mainly used for advanced PD patients (Car et al., 2019) and has not yet been marketed for PD in Mainland China. Therefore, this study focused on six NEDAs available for clinical use in Mainland China, with the scoring order of pramipexole ER tablets (Sifrol), pramipexole IR tablets (Sifrol), rotigotine patch (Neupro), piribedil tablets (Trastal), ropinirole PR tablets (Requip), and ropinirole IR tablets (Requip). The specifications of the six NEDAs were selected based on those commonly used in clinical practice (Table 1).

**TABLE 1:** The manufacturers and specifications of six drugs.

Drug	Manufacturers	Specifications
Pramipexole ER	Boehringer Ingelheim Pharma GmbH & Co. KG	0.75 mg
Pramipexole IR	Boehringer Ingelheim Pharma GmbH & Co. KG	0.25 mg
Rotigotine patch	LTS Lohmann Therapie- Systeme AG	6 mg
Piribedil	LES LABORATOIRES SERVIER INDUSTRIE	50 mg
Ropinirole PR	GLAXO WELLCOME, S.A.	2 mg
Ropinirole IR	GLAXO WELLCOME, S.A.	0.5 mg

Abbreviations: ER, extended-release; IR, immediate-release; PR, prolonged-release.

### 2.2 Evaluation framework and scoring methodology

This study employed the Rapid Guide for Drug Evaluation and Selection in Chinese Medical Institutions (Second Edition) (hereinafter referred to as “the Selection Guidelines”) (Zhao et al., 2023), which integrates the rapid HTA with the Structured Operational Judgment Approach (SOJA) framework. The evaluation dimensions and their corresponding weights were established through a rigorous, multi-round, modified Delphi consensus process detailed within the guideline.

The six NEDAs were assessed across five predefined dimensions using a validated MCDA tool. The MCDA methodology serves as the core analytical approach of this study, designed to synthesize complex, multi-dimensional evidence into a structured decision-support tool. Its analytical rigor is derived from the transparency of its structure and the systematic application of pre-defined criteria. For each sub-criterion within these dimensions, scoring was conducted by applying explicit, evidence-based rules predefined in the Selection Guidelines. All scores were derived from a systematic review of clinical trial data, regulatory documents, and high-quality meta-analyses. It is important to note that while expert opinion was incorporated into the pre-defined Delphi-derived framework, it was not used directly to assign scores to individual drugs. The complete scoring matrix, including detailed criteria, evidence sources, and score justifications, is provided in Supplementary Table S1.

### 2.3 Retrieval and evaluation of relevant evidence

A systematic literature search was conducted across international and Chinese databases—including PubMed, Embase, CNKI, and Wanfang—as well as official regulatory and health organization websites such as the National Medical Products Administration (NMPA), the World Health Organization, the U.S. Food and Drug Administration (FDA), and Yaozhiwang. The search encompassed the period from database inception to June 2024 and aimed to identify evidence on the six NEDAs, covering indications, pharmacological characteristics, guideline recommendations, drug registration status, pricing, and market information. The search strategies for PubMed is provided in Supplementary Table S1. Literature screening, data extraction, and evaluation were performed independently by two pharmacists. Any discrepancies were resolved through discussion or by consulting a third expert when necessary.

The inclusion criteria were as follows: (1) the article was a RCT or meta-analysis; with the language of articles limited to Chinese and English; (2) the study population was early PD patients; (3) the studies compared pramipexole ER, pramipexole IR, rotigotine patch, piribedil, ropinirole PR, ropinirole IR with other therapeutic drugs, placebo, or no drug; (4) the efficacy indicators: unified Parkinson’s Disease Rating Scale-Activities of daily living (UPDRS-II) for activities of daily living, Unified Parkinson’s Disease Rating Scale-Motor (UPDRS-III) for motor function, and Unified Parkinson’s Disease Rating Scale—motor function and activities of daily living (UPDRS-II + III) scores; and (5) the safety indicators: adverse events, serious adverse events and death.

The exclusion criteria were as follows: (1) study design included case reports, reviews, retrospective studies; (2) duplicate studies; (3) literature with unavailable data.

### 2.4 Analytical rigor and sensitivity considerations

To ensure the robustness of our evaluation, we implemented several methodological safeguards. Data extraction and preliminary scoring were performed independently by two researchers to minimize potential bias. A predefined adjudication process was established whereby any scoring discrepancy exceeding 3 points in any dimension triggered review by a third independent expert, with final scores determined through consensus. This process enhanced scoring consistency and reduced subjectivity. The foundation of our scoring system relies on high-quality evidence sources. Specifically, the comparative efficacy and safety data were derived from network meta-analyses that employed rigorous statistical methods, including Bayesian frameworks and random-effects models. These methods inherently account for variance and uncertainty in the input data, thereby providing a statistically robust foundation for our subsequent scoring.

Composite scores were categorized into three distinct recommendation levels: Strong Recommendation (≥70 points), Conditional Recommendation (60–69 points), and Non-Recommendation (<60 points). This approach ensures that drugs within the same recommendation tier are considered clinically equivalent, regardless of minor score variations, thereby focusing interpretation on clinically meaningful differences.

## 3 Results

### 3.1 Pharmacological properties score

#### 3.1.1 Pharmacological effects

According to the instructions and relevant literature (Jing et al., 2023; National Medical Products Administration, 2015a; National Medical Products Admi nistration, 2017; National Medical Products Administration, 2018; National Medical Products Administration, 2013; National Medical Products Administration, 2015b; National Medical Products Administration, 2016; Antonini et al., 2010; Boll et al., 2011; Elshoff et al., 2015; Millan, 2010; Contin et al., 2019), the pharmacological effects of the six drugs were scored. The Chinese guideline (Chen, 2020) and Canadian guideline (Grimes et al., 2019) for the PD recommend NEDAs as the preferred treatment for early PD. As shown in Table 2, the clinical efficacy of the rotigotine patch is definite, but the specific mechanisms are still unclear, with a pharmacological effect score of 2 points. The other five drugs had definite clinical efficacy and clear pharmacological effects but lacked innovation in their mechanisms or targets, with a score of 4 points.

**TABLE 2:** The pharmacological effects of six drugs (National Medical Products Administration, 2016; Antonini et al., 2010; Boll et al., 2011; Elshoff et al., 2015; Millan, 2010).

Drug	Mechanism of action	Pharmacological effects score
Pramipexole ER (National Medical Products Administration, 2015a)	It acts on dopamine D2/D3 receptors, and its binding ability to dopamine D3 receptors is 7–10 times that of D2 receptors	4
Pramipexole IR (National Medical Products Admi nistration, 2017)		4
Rotigotine patch (National Medical Products Administration, 2018)	The mechanism of action is still unclear, possibly due to its high affinity for dopamine D1, D2, and D3 receptors, but low affinity for dopamine D4 and D5 receptors	2
Piribedil ER (National Medical Products Administration, 2013)	It has a strong affinity for dopamine D2/D3 receptors, but a low affinity for α2-adrenergic and 5-hydroxytryptamine (5-HT) receptors	4
Ropinirole PR (National Medical Products Administration, 2015b)	It stimulates dopamine D2 and D3 receptors, but has almost no affinity for dopamine D1, adrenergic, and serotonergic receptors	4
Ropinirole IR (National Medical Products Administration, 2016)		4

Abbreviations: ER, extended-release; IR, immediate-release; PR, prolonged-release.

#### 3.1.2 Pharmacokinetics

The six NEDAs were scored on their *in vivo* profiles according to established data on their absorption, distribution, metabolism, and excretion in the humans. As the pharmacokinetic parameters described in the drug instruction and relevant literature (Poewe et al., 2011; Boll et al., 2011) were well-defined, all drugs received a score of 5 points (Table 3).

**TABLE 3:** The pharmacokinetic score of the six drugs.

Drug	Pro-drug	Drug delivery route	F/%	t_max_/h	PPBR/%	t_1/2_/h	CL/(L·h^-1^)	Metabolic enzyme	Renal clearance/%	Pharmacokinetic score
Pramipexole ER (National Medical Products Administration, 2015a)	—	oral	100.00	6.00	15.00	8.00–12.00	66.67	—	90.00	5
Pramipexole IR (National Medical Products Admi nistration, 2017)	—	oral	90.00	2.00	15.00	8.00–12.00	66.67	—	80.00	5
Rotigotine patch (National Medical Products Administration, 2018)	—	external use	37.00	15.00–18.00	89.50	5.00–7.00	300.00–600.00	CYP isoenzymes	71.00	5
Piribedil ER (National Medical Products Administration, 2013)	—	oral	—	1.00	—	1.70–6.90	—	—	68.00	5
Ropinirole PR (National Medical Products Administration, 2015b)	—	oral	100.00	6.00–10.00	40.00	6.00	47.00	CYP1A2	88.00	5
Ropinirole IR (National Medical Products Administration, 2016)	—	oral	85.00	1.00–2.00	40.00	6.00	47.00	CYP1A2	88.00	5

Abbreviations: F, relative bioavailability; tmax, time to peak; PPBR, plasma protein binding rate; t_1/2_, plasma half life; CL, clearance; CYP, cytochrome P; CYP1A2, cytochrome P1A2; ER, extended-release; IR, immediate-release; PR, prolonged-release.

#### 3.1.3 Pharmaceutical formulation and administration, storage conditions

The pharmaceutical formulation and administration, storage conditions of the six NEDAs were evaluated. All six drugs achieved consistently high scores in terms of ingredient simplicity, specification suitability, and self-administration capability. However, substantial variations were observed in their administration frequency and storage requirements As detailed in Table 4, the total scores for these parameters across the six NEDAs were 17, 15, 17, 15, 16.5, and 14.5, respectively.

**TABLE 4:** The pharmaceutical formulation and administration, storage conditions of six drugs.

Drug	Pharmaceutical formulation and administration (12 points)						Storage conditions (6 points)		Total score
	Main ingredients and excipients (2 points)	Specifications and packaging (2 points)	Dosage form (2 points)	Dosage (2 points)	Administration frequency (2 points)	Convenient (2 points)	Storage conditions (4 points)	Drug shelf life (2 points)	
Pramipexole ER	Pramipexole dihydrochloride monohydrate、hypromellose, corn starch, carbomer homopolymer, etc. (2 points)	Suitable for clinical application/dosage adjustment. (2 points)	Tablet (2 points)	Dosage adjustment is required during use (1.5 points)	Once a day (2 points)	Can be self-administered without assistance (Dorsey and Bloem, 2018)	Sealed storage at room temperature (4 points)	36 months (1.5 points)	17
Pramipexole IR	Pramipexole dihydrochloride monohydrate, corn starch, microcrystalline cellulose, etc. (2 points)		Tablet (2 points)		Three times a day (1 points)		Sealed storage below 30 °C in the dark (3 points)	36 months (1.5 points)	15
Rotigotine patch	Rotigotine, ascorbyl palmitate, povidone, silicone adhesive, sodium metabisulfite, and dl-alpha-tocopherol. (2 points)		Patch (2 points)		Once a day (2 points)		Storage below 30 °C (4 points)	30 months (1.5 points)	17
Piribedil ER	Piribedil, fructose, etc. (2 points)		Tablet (2 points)		Three-five times a day (1 points)		Store in a light shielded and sealed environment (3 points)	36 months (1.5 points)	15
Ropinirole PR	Ropinirole hydrochloride, ethylcelluloses, ferric oxide red, maltodextrin, etc. (2 points)		Tablet (2 points)		Once a day (2 points)		Store in a dry place below 25 °C (4 points)	24 months (1 points)	16.5
Ropinirole IR	Ropinirole hydrochloride, lactose monohydrate, magnesium stearate, etc. (2 points)		Tablet (2 points)		Three times a day (1 points)		Store in a cool, dark and sealed place (3 points)	24 months (1 points)	14.5

Abbreviations: etc, etcetera; ER, extended-release; IR, immediate-release; PR, prolonged-release; etc, etcetera.

The pharmacological properties of the six drugs—covering pharmacological effects, pharmacokinetics, pharmaceutical formulation and administration, and storage conditions—were comprehensively assessed. Based on this evaluation, pramipexole ER achieved the highest score, whereas ropinirole IR received the lowest.

### 3.2 Efficacy score

#### 3.2.1 Indications

To explore the clinical application of the six drugs in the treatment of diseases, their indications were evaluated. Based on drug instructions and related research (Chen, 2020; Grimes et al., 2019; National Medical Products Administration, 2015a; National Medical Products Admi nistration, 2017; National Medical Products Administration, 2018; National Medical Products Administration, 2013; National Medical Products Administration, 2015b; National Medical Products Administration, 2016), all six drugs are recommended as first-line drugs for early PD and were rated as clinically essential, each achieving the maximum score of 3.

#### 3.2.2 Guideline recommendations

By referring to the recommendations and evidence levels of six NEDAs in the guidelines and expert consensus, the associated recommendations were assessed to determine the clinical application value of six NEDAs (Su and Chen, 2021; Chen, 2020; Fox et al., 2018; Grimes et al., 2019; Ferreira et al., 2013). As shown in Table 5, most NEDAs received highest scores based on strong guideline recommendations and evidence-based medical evidence, while ropinirole PR scored lower due to weaker support from the guidelines.

**TABLE 5:** Guide recommendation of six drugs.

Guide	Pramipexole ER	Pramipexole IR	Rotigotine transdermal patch	Piribedil ER	Ropinirole PR	Ropinirole IR
Evidence based medicine guidelines for the treatment of early motor symptoms of Parkinson’s disease in China regarding the treatment of early motor symptoms (Su and Chen, 2021)	1A	1A	1A	1A	1B	1A
Evidence based medicine guidelines for the treatment of early motor symptoms in Parkinson’s disease in China regarding the prevention/delay of motor complications (Su and Chen, 2021)	1A	1A	—	—	1B	1B
Chinese Parkinson’s disease Treatment Guidelines (Fourth Edition) regarding the treatment of early Parkinson’s disease (Chen, 2020; Fox et al., 2018)	1	1	1	1	1	1
The 2013 edition of the European Federation of Neurology (EFNS) guidelines regarding the treatment of early Parkinson’s disease (Ferreira et al., 2013)	1A	1A	1A	1C	1A	1A
Canadian guideline for Parkinson disease 2019 regarding the treatment of early Parkinson’s disease (Grimes et al., 2019)	A	A	A	A	A	A
Guide recommendation score (12 points)	12	12	12	12	8	12

1A, guideline level I recommendation, high quality evidence; 1B, guideline level I recommendation, moderate quality evidence.

Abbreviations: ER, extended-release; IR, immediate-release; PR, prolonged-release.

#### 3.2.3 Clinical efficacy

The clinical efficacy of the six NEDAs was evaluated based on clinical trials and systematic reviews (Chen et al., 2023; Poewe et al., 2011; Giladi et al., 2007), using the UPDRS-Ⅱ as the primary indicator and the UPDRS-III, UPDRS-II + III as secondary indicators. A meta-analysis (Chen et al., 2023) found that while all six NEDAs significantly improved UPDRS-II, UPDRS-III, and UPDRS-II + III scores compared to placebo, no statistically significant differences were observed among them in UPDRS-II and UPDRS-III scores. However, for the UPDRS-II + III score, ropinirole IR/PR and piribedil demonstrated superior improvement over the rotigotine patch. Furthermore, piribedil showed the greatest improvement in UPDRS-II and UPDRS-III scores. Based on the body of evidence summarized in Table 6, the six NEDAs received clinical efficacy scores of 7, 7, 6, 10, 9, and 8, respectively.

**TABLE 6:** Clinical efficacy of six drugs.

References	Study type	Clinical efficacy conclusion
Poewe (Poewe et al., 2011)	Randomized controlled trial	Among 213 patients for pramipexole ER and 207 patients for pramipexole IR recipients, the adjusted mean 33-week UPDRS II + III change (excluding levodopa rescue effects) was −8.2 for pramipexole ER and −8.7 for pramipexole IR. Compared with placebo (n = 103), pramipexole ER and pramipexole IR were significantly superior on UPDRS II + III score, all key secondary outcomes, and almost all other endpoints
Giladi (Giladi et al., 2007)	Randomized controlled trial	Patients (n = 561) were randomized to rotigotine, ropinirole, or placebo. The primary endpoint was the proportion of patients with a minimum of 20% decrease in UPDRS-II + III scores. The responder rate in the rotigotine group was significantly higher than in the placebo group (52% vs. 30%, P < 0.0001). Rotigotine at doses < or = 8 mg/24 h did not show noninferiority to ropinirole at doses < or = 24 mg/day
Chen (Chen et al., 2023)	System Review and Meta Analysis	A total of 20 Randomized clinical trials (5,355 patients) were included in the study. There were no statistically significant differences between six NEDAs for the UPDRS-II and UPDRS-III. For UPDRS-II + III, the improvement of ropinirole IR/PR and piribedil were higher than that of rotigotine transdermal patch, and piribedil piribedil and ropinirole PR exhibited similar improvement

Abbreviations: ER, extended-release; IR, immediate-release; PR, prolonged-release; UPDRS-II, unified PD, rating Scale-Activities of daily living; UPDRS-III, unified PD, rating scale-motor; UPDRS-II + III, unified PD, rating scale-motor function and activities of daily living.

In summary, in the evaluation of clinical efficacy, piribedil was the most effective (25 points), followed by ropinirole IR (23 points), pramipexole ER (22 points), pramipexole IR (22 points), rotigotine patch (21 points), and ropinirole PR (20 points).

### 3.3 Safety score

#### 3.3.1 Adverse reactions

The incidence rate of adverse reactions to the six NEDAs in the drug instructions was explored and scored. The mild and moderate adverse effects of the six NEDAs included nausea, drowsiness, dizziness, headache and so on, and their incidence rates were greater than 10%. In a phase III trial evaluating the treatment of PD in Chinese patients with pramipexole ER and pramipexole IR, the most common drug-related adverse reaction rates for two drugs were drowsiness (ER: 18.8%, IR: 14.6%), dizziness (ER: 11.5%, IR: 12.1%), and nausea (ER: 7.3%, IR: 6.3%) (Wang et al., 2014). Impulsive control disorder is one of the serious adverse reactions of NEDAs (Jing et al., 2023), with an incidence rate of 1%–10% in pramipexole ER, pramipexole IR, rotigotine patch, and piribedil, whereas the incidence rates of ropinirole PR and ropinirole IR are 0.1%–1%. As shown in Table 7, Both ropinirole formulations (PR and IR) were ranked higher in terms of adverse reactions scores than the other five drugs.

**TABLE 7:** The adverse reactions score in six drugs.

Drug	Moderate adverse reactions and incidence rate (3 points)	Severe adverse reactions and incidence rate (5 points)	Score (8 points)
Pramipexole ER (National Medical Products Administration, 2015a)	≥10%: Gastrointestinal (nausea), nervous system (drowsiness, dizziness, headache), autonomic nervous system disorders (constipation) (1 points)	1%–10%: impulse control disorder (2 points)	3
Pramipexole IR (National Medical Products Admi nistration, 2017)			3
Rotigotine patch (National Medical Products Administration, 2018)			3
Piribedil (National Medical Products Administration, 2018)			3
Ropinirole PR (National Medical Products Administration, 2013)		0.1%–1%: impulse control disorder (3 points)	4
Ropinirole IR (National Medical Products Administration, 2015b)			4

Abbreviations: ER, extended-release; IR, immediate-release; PR, prolonged-release.

#### 3.3.2 Special groups

The use of six NEDAs in special populations, including children, the elderly, pregnant and lactating women, and patients with liver or renal impairment, was studied to evaluate their safety. Ropinirole PR/IR is classified as grade C pregnancy. It can only be taken during pregnancy when the potential benefits to the fetus outweigh the potential risks, and cannot be taken during lactation, with a score of 0.5. Owing to the lack of safety studies in pediatric patients and the potential drug exposure risks to pregnant women and lactating fetuses, the other four drugs were not recommended for use in these patients. For patients with liver failure, adjusting the dosage of pramipexole ER, pramipexole IR, and piribedil is not recommended, and the use of rotigotine patch should be cautious in cases of severe hepatic dysfunction. However, ropinirole PR and ropinirole IR are not recommended for patients with hepatic dysfunction. No dosage adjustment is required for pramipexole ER, pramipexole IR, ropinirole PR and ropinirole IR in patients with mild or moderate renal dysfunction. For patients with severe renal dysfunction, adjusting the dosage of rotigotine patch is unnecessary, but dosage modification is essential for piribedil. Therefore, the special groups scores for the six NEDAs were 6, 6, 6, 5, 3.5, 3.5, respectively, as shown in Table 8.

**TABLE 8:** Special groups score of six drugs.

Drug	Pediatric use (2 points)	Geriatric use (1 points)	Pregnant women (1 points)	Lactating women (1 points)	Liver impairment (3 points)	Renal impairment (3 points)	Total score (11 points)
Pramipexole ER	0	1	0	0	3	2	6
Pramipexole IR	0	1	0	0	3	2	6
Rotigotine patch	0	1	0	0	2	3	6
Piribedil	0	1	0	0	3	1	5
Ropinirole PR	0	1	0.5	0	0	2	3.5
Ropinirole IR	0	1	0.5	0	0	2	3.5

Abbreviations: ER, extended-release; IR, immediate-release; PR, prolonged-release.

#### 3.3.3 Drug interactions and other safety score

This study was conducted to explore the interactions of six NEDAs with other drugs and assess the reversibility of their adverse reactions, teratogenicity, carcinogenicity, and additional safety factors. Drug interactions and the safety of various drugs have been explained in drug instructions. This suggests that the co-administration of pramipexole and some drugs eliminated through renal secretion (such as cimetidine, ranitidine, and diltiazem) decreases the oral clearance rate by approximately 20%, scoring 2 points. Interaction between the rotigotine patch and other drugs is not observed, scoring 3 points, while piribedil is prohibited from being used in combination with antiemetic antipsychotics and dopaminergic agonists, earning 1 point. Cytochrome P1A2 (CYP1A2) is the main enzyme involved in ropinirole metabolism. It may be necessary to adjust the dosage of ropinirole during the initiation or withdrawal of CYP1A2 inducers or inhibitors. Moreover, the adverse reactions caused by the six drugs are reversible, with a score of 1.

In terms of teratogenicity and carcinogenicity, all six drugs received a score of 0. Animal studies have not shown any teratogenic effects of pramipexole ER/IR and rotigotine patch in rats or rabbits, but embryotoxicity has been observed in rats and mice at maternal toxicity dose levels (National Medical Products Administration, 2015a; National Medical Products Admi nistration, 2017; National Medical Products Administration, 2018). There has been no corresponding research on piribedil. Adverse effects on embryonic development, including teratogenic effects, are observed with ropinirole PR/ER. In addition, there are black warnings in the FDA and NMPA for pramipexole ER, pramipexole IR, and ropinirole IR with a score of 0; however, drug warnings have not yet been issued for the other three drugs with a score of 1. The drug interactions and other safety score for the six NEDAs were 3, 3, 5, 3, 4, and 3, respectively. The specific evaluation scores for the six drugs are listed in Table 9.

**TABLE 9:** Drug interactions and other safety score of six drugs.

Drug	Drug interaction score (3 points)	Other safety score (3 points)	Total score (6 points)
Pramipexole ER	The combination of pramipexole and some drugs cleared through the renal pathway should consider reducing the dosage (2 points)	Reversible adverse reactions (1 points), embryotoxicity (0 points), no special drug warning (0 points)	3
Pramipexole IR			3
Rotigotine patch	No interaction with other drugs (3 points)	Reversible adverse reactions (1 points), embryotoxicity (0 points), no special drug warning (1 points)	5
Piribedil	Prohibition of combined use with antiemetic psychotropic drugs, dopaminergic agonists, etc (1 points)		3
Ropinirole PR	When used in combination with potent CYP1A2 inhibitors, the dosage should be adjusted (2 points)	Reversible adverse reactions (1 points), teratogenic (0 points), no special drug warning (1 points)	4
Ropinirole IR		Reversible adverse reactions (1 points), teratogenic (0 points), and have special drug warnings (0 points)	3

Abbreviations: ER, extended-release; IR, immediate-release; PR, prolonged-release; CYP1A2, cytochrome P1A2.

Therefore, the safety scores for pramipexole ER, pramipexole IR, rotigotine patch, piribedil, ropinirole PR, and ropinirole IR were 12 points, 12 points, 14 points, 11 points, 10.5 points, and 11.5 points, respectively.

### 3.4 Economy score

The recommended starting dose and unit price of the six drugs were obtained from drug instructions or official websites such as yaozhi (bd.yaozh.com). Based on the differences in daily treatment costs between the six drugs and generic drugs as well as alternative drugs with main indications, their economics were scored to provide evidence for pharmacoeconomic research (Ji et al., 2025). As shown in Table 10, piribedil demonstrated the most favorable economic profile, while ropinirole IR ranked lowest in economic evaluation.

**TABLE 10:** Economy score of six drugs.

Drug	Average daily treatment cost (RMB)^a^	Economy (10 points)		Total score
		Score for the same generic name^b^ (3 points)	Score for alternative drugs with the main indications^c^ (7 points)	
Pramipexole ER	7.82	0.81	6.38	7.19
Pramipexole IR	7.82	0.22	6.38	6.60
Rotigotine patch	9.28	3.00	5.38	8.38
Piribedil	7.13	3.00	7.00	10.00
Ropinirole PR	9.96	2.55	5.01	7.56
Ropinirole IR	17.63	2.08	2.83	4.91

a the average daily treatment cost is calculated based on the unit price and recommended starting dose of drugs; b, The scoring criteria for drugs with the same generic name: The drug with the lowest average daily treatment cost is scored 3 points, and the evaluation drug score = the lowest daily treatment cost/average daily treatment cost of the evaluated drug ×3; c, Rules for scoring alternative drugs with major indications: The drug with the lowest daily treatment cost is scored 7 points, and the evaluation drug score = the lowest daily treatment cost/average daily treatment cost of the evaluated drug ×7.

Abbreviations: ER, extended-release; IR, immediate-release; PR, prolonged-release.

### 3.5 Other attributes score

The other attributes of the six NEDAs were scored across multiple parameters including insurance coverage, essential medicine status, procurement inclusion, and global availability. Pramipexole ER, pramipexole IR, and piribedil were included in category B reimbursement of Chinese National Health Insurance Catalog, with no payment limitations and a score of 2 points, while ropinirole PR/IR have payment limitations, with 1.5 points. Rotigotine patch was not included in the national health insurance catalog and was scored 1 point. According to whether the six drugs are entered in Chinese national essential medicines list and national drug centralized procurement list, their scores for national essential medicines were 2, 2, 1, 1, 1, 1, and the scores for national drug centralized procerement were 1, 1, 0, 0, 0 and 0, respectively. Six NEDAs were original drugs and all scored 1 point.

The scores for pharmaceutical manufacturers were determined based on the 2023 rankings of global pharmaceutical companies. Among them, the manufacturers of pramipexole ER, pramipexole IR, rotigotine patch, piberpidil, ropinirole ER, and ropinirole IR ranked 16th, 16th, 31st, 34th, 10th and 10th, respectively, with scores of 0.8, 0.8, 0.4, 0.4, 1, and 1, respectively. In addition, six drugs were scored based on global drug usage. Piribedil was marketed in China and Europe but not in the United States or Japan, with a score of 0.5. The other five drugs were marketed in China, Japan, the United States, Europe, and other countries with a score of 1.

As detailed in Table 11, among the various attribute scores, pramipexole ER and pramipexole IR exhibited the highest scores, whereas the rotigotine patch received the lowest score.

**TABLE 11:** Other attributes score of six drugs.

Drug	Other attributes (10 points)						Total score (10 points)
	Score of national health insurance (3 points)	Score of national essential drug (3 points)	Score of national centralized drug procurement (1 points)	Score of the original drug (1 points)	Score of production enterprise ranking (1 points)	Score of global usage (1 points)	
Pramipexole ER	In the national health insurance catalog category B reimbursement with no payment limitations (2 points)	As a national essential drug, there are △ requirements (2 points)	Yes (1 points)	Original drug (1 points)	16th (0.8 points)	1	7.8
Pramipexole IR			Yes (1 points)		16th (0.8 points)	1	7.8
Rotigotine patch	No in the national health insurance catalog (1 points)	Not a national essential drug (1 points)	No (0 points)		31st (0.4 points)	1	3.4
Piribedil	In the national health insurance catalog category B reimbursement with no payment limitations (2 points)		No (0 points)		34th (0.4 points)	0.5	4.9
Ropinirole PR	In the national health insurance catalog category B reimbursement with payment limitations (1.5 points)		No (0 points)		10th (1 points)	1	5.5
Ropinirole IR			No (0 points)		10th (1 points)	1	5.5

Abbreviations: ER, extended-release; IR, immediate-release; PR, prolonged-release; △, it means that drugs should be used by physicians with corresponding prescription qualifications or under the guidance of specialist physicians, and monitoring and evaluation of their use should be strengthened.

### 3.6 Comprehensive score

Based on the 5 dimensions of pharmacological properties, efficacy, safety, economy, and other attributes, the comprehensive scores of pramipexole ER, pramipexole IR, rotigotine patch, piribedil, ropinirole PR and ropinirole ER were 74.99, 72.40, 70.78, 74.90, 69.06, and 68.41, respectively, as shown in Table 12. The comprehensive evaluation results demonstrated that pramipexole ER had the highest score, while ropinirole IR had the lowest.

**TABLE 12:** The comprehensive score of six drugs.

Drug	Pharmacological properties	Efficacy	Safety	Economy	Other attributes	Comprehensive score
Pramipexole ER	26	22	12	7.19	7.8	74.99
Pramipexole IR	24	22	12	6.60	7.8	72.40
Rotigotine patch	24	21	14	8.38	3.4	70.78
Piribedil	24	25	11	10.00	4.9	74.90
Ropinirole PR	25.5	20	10.5	7.56	5.5	69.06
Ropinirole IR	23.5	23	11.5	4.91	5.5	68.41

Abbreviations: ER, extended-release; IR, immediate-release; PR, prolonged-release.

## 4 Discussion

### 4.1 Comparison with existing economic and clinical evidence

Nowadays, there are a variety of NEDAs for symptomatic treatment of PD, but there is little information on how these options compare comprehensively. Our study employed a rapid HTA framework to fill this gap. We identified four drugs—pramipexole ER, pramipexole IR, rotigotine patch, and piribedil—that achieved scores above 70 points, warranting their strong recommendation for inclusion in the formularies of medical institutions.

To contextualize and validate these findings within the broader evidence base, we conducted a systematic comparison with existing pharmacoeconomic and clinical literature, particularly relevant given the absence of comprehensive HTA reports for all six NEDAs. Our strong recommendation for pramipexole formulations is consistently supported by international evidence, which confirms their superior cost-effectiveness profile compared to other drugs (Noyes et al., 2005; Hoerger et al., 1998; Jiang, 2020). Furthermore, the recommendation for rotigotine patches, despite their higher economic cost, finds support in international clinical assessments that acknowledge the unique therapeutic value of transdermal delivery for specific patient populations (Sanford and Scott, 2011; Boroojerdi et al., 2010). Similarly, the prominent positioning of piribedil in our assessment reflects the importance of regional treatment contexts. While formal, head-to-head cost-effectiveness analyses comparing piribedil to other NEDAs are scarce in the international literature, its favorable evaluation in our model is strongly supported by its low acquisition cost within the Chinese healthcare system and by compelling efficacy data from network meta-analyses (Chen et al., 2023). This evidence collectively positions it as a cost-efficient alternative for formulary inclusion, particularly in resource-conscious settings. Therefore, our study addresses a critical evidence gap by providing a comprehensive head-to-head comparison within a unified framework. Crucially, this integrated assessment yields conclusions that are not novel in isolation but are robustly corroborated by the existing body of credible evidence, thereby validating the reliability of our methodological approach.

### 4.2 Analysis of individual drug profiles and scores

Pramipexole ER had the highest comprehensive score, attributing to its excellent pharmaceutical properties, efficacy, and other attributes compared with the other five drugs (Hametner et al., 2011). A study found that dopamine agonists, in particular pramipexole, were effective and safe as monotherapy in managing symptoms of PD (Binde et al., 2020). Piribedil is one of the first NEDAs to be marketed and widely used as an extended-release oral formulation in European, Latin American, and Asian countries (Perez-Lloret and Rascol, 2016). However, it scored slightly lower than pramipexole ER due to more frequent daily dosing (3–5 times/day), specific storage requirements, and limited inclusion in essential medicine lists outside certain countries. Owing to the high frequency of drug use, pramipexole IR scored slightly lower in terms of its pharmaceutical characteristics. However, in terms of clinical efficacy, piribedil had a better clinical efficacy than pramipexole ER. The rotigotine patch ranked fourth among the six drugs, partly due to the absence of generic versions in China, an unclear mechanism of action, and lack of medical insurance coverage. Ropinirole PR/IR received the lowest evaluation scores, with low ratings noted particularly in terms of efficacy, safety, and economic factors. Clinical evidence supporting ropinirole is relatively limited, and guidelines classify it as a second-line option for early PD (Chen, 2020). It is not recommended for patients with liver impairment due to potential teratogenic effects, and studies have associated it with a higher risk of adverse events compared to other NEDAs (Chen et al., 2023; Li et al., 2017). Furthermore, due to their high prices and the absence of generic drugs in China, their economic scores were relatively low.

Therefore, physicians can make reasonable drug choices for patients with early PD based on the scores of the six NEDAs and recommendations from the guidelines in this study, as well as the clinical diagnosis and performance of patients. Based on pharmacological characteristics and approved labeling, pramipexole ER/IR and piribedil are considered suitable for patients with hepatic dysfunction, whereas ropinirole PR/IR is not recommended in this population. Black box warnings have been issued for both pramipexole ER/IR and ropinirole IR, highlighting serious risks that require careful consideration. Furthermore, it is critical to emphasize that the clinical performance and safety profiles of these NEDAs in special populations (such as children, and pregnant or lactating women) remain unestablished. Consequently, the scoring results do not apply to these populations. Future targeted clinical studies are urgently required to generate evidence that can reliably inform clinical decision-making for these vulnerable patients.

The multi-dimensional evaluation framework developed in this study provides an actionable tool to guide drug selection and formulary management for NEDAs within hospitals. For hospital pharmacy and therapeutics committees, this rapid HTA model enables a standardized and transparent process for formulary management. By quantitatively comparing drugs across efficacy, safety, insurance coverage, and procurement accessibility, the model provides an evidence-based rationale for formulary inclusion, positioning, and procurement planning. It helps institutions rapidly identify drugs that are clinically effective, policy-aligned, and economically efficient, thereby optimizing resource allocation. For clinicians, the structured scoring system yields clear, comparative insights to support precise drug selection. It supports tailored treatment regimens for individual patients by elucidating differences in pharmacological properties and safety profiles, thus promoting personalized and efficient therapeutic choices. It is important to note that this multi-dimensional scoring system is dynamic. Such real-time adjustments will ensure that the tool continues to provide reliable, timely evidence for hospital decision-makers in a rapidly evolving healthcare environment.

### 4.3 Transferability and dynamic nature

This study develops a rapid and comprehensive scoring tool for evaluating six NEDAs in early PD treatment, based on ‘the selection guidelines’. While conceived within the Chinese healthcare context, the model is designed with transferability as a core principle, providing a structured framework for pharmaceutical evaluation and selection by medical institutions globally. To ensure its application in other countries or regions, we propose the following actionable guidance: (1) Adopt universally applicable core dimensions: The core dimensions of pharmacological characteristics, efficacy, and safety are of universal relevance, and their evaluation standards and initial weight criteria can be directly adopted in most settings to maintain clinical and scientific rigor. (2) Customize context-specific indicators: As indicators such the essential medicines list, the national medical insurance catalog, and centralized drug procurement exhibit distinct Chinese characteristics, evaluators should replace these with locally relevant metrics (e.g., national formulary status, or procurement policies) to align the model with local health policies and healthcare structures. (3) Calibrate criterion weights via expert Consensus: The weight of each criterion must be recalibrated to reflect local priorities, policies, and resource constraints. We recommend employing a formal consensus method, such as the Delphi technique, with a panel of local clinical, pharmacological, and health economic experts to resolve controversies and establish validated local weights. (4) Implement a structured dynamic update mechanism: We recommend conducting comprehensive reassessments every two or 3 years, complemented by immediate updates triggered by major developments including new drug approvals, publication of pivotal clinical evidence, significant guideline updates, substantial policy changes, or major price alterations. All updates should be executed by a convened multidisciplinary expert panel using the original MCDA framework to maintain methodological consistency and ensure the model remains a reliable, current decision-support tool in the evolving healthcare landscape.

### 4.4 Limitations

This study provides health decision-makers with timely scientific options or decisions on rationally applying health technologies and allocating health resources. However, several limitations should be noted. Firstly, the absence of real-world data means our findings are primarily based on efficacy under controlled trial conditions, which may not fully represent effectiveness in routine clinical practice with more diverse patient populations. Secondly, the time-sensitive nature of rapid HTA means scores may change with updates to guidelines, policies, or drug prices. To address these limitations, future research should integrate real-world evidence from large-scale registries and electronic health records, and establish a structured process for dynamically updating the assessment as major new evidence and policies emerge.

## 5 Conclusion

In summary, this study identifies pramipexole ER, pramipexole IR, rotigotine patch, and piribedil are strongly recommended for inclusion in medical institution formularies for treating early PD. The evaluation framework provides a key tools and evidence for drug selection and clinical rational use of PD patients in medical institutions, while also offering a transferable methodology for healthcare systems in other regions.

## Data Availability

The original contributions presented in the study are included in the article/Supplementary Material, further inquiries can be directed to the corresponding authors.
