# Supplementary material for: Rapid health technology assessment of six non-ergot dopamine-receptor agonists for the treatment of early Parkinson’s disease
Source: Front Pharmacol. 2025 Dec 17;16:1648833. doi: 10.3389/fphar.2025.1648833 (PMC12753440; doi:10.3389/fphar.2025.1648833)
Supplement: Supplementary file 1 [file Supplementaryfile1.docx]

**Supplementary Table 1 Evaluation dimensions, weights, and standardized scoring criteria (Zhao et al., 2023)**

| **Evaluation dimensions and corresponding weights （100 points）** | | | **Scoring criteria** |
| --- | --- | --- | --- |
| **Pharmaceutical properties (28 points)** | Pharmacological  effects (5 points) | Definite clinical efficacy, precise mechanism of action, and innovative mechanism of action or target point of action | 5 |
|  |  | Definite clinical efficacy and precise mechanism of action | 4 |
|  |  | Fair clinical efficacy and mechanism of action are unclear | 2 |
|  |  | General clinical efficacy and unclear mechanism of action | 1 |
|  | Pharmacokinetics (5 points) | Well-defined in vivo process with complete pharmacokinetic parameters | 5 |
|  |  | Well-defined in vivo process with incomplete pharmacokinetic parameters | 3 |
|  |  | In vivo processes are unclear, or no pharmacokinetic studies are available | 1 |
|  | Pharmaceutical formulation and administration (multiple choice)  (12 points) | Main ingredients and excipients (all specify 2; one specify 1) | 2 |
|  |  | Specification and packaging (all appropriate for clinical use/dose adjustment 2; one appropriate 1) | 2 |
|  |  | Dosage forms (oral/inhalation/topical formulations 2; subcutaneous/intramuscular injections 1.5; intravenous drip/intravenous injections 1) | 2 |
|  |  | The dose administered (fixed dose 2; dose to be adjusted during use 1.5; dose based on body mass or body surface area 1) | 2 |
|  |  | Frequency of administration (<1 dose/d 2; 2 doses/d 1.5; ≥3 doses/d 1) | 2 |
|  |  | Ease of use (self- administration without assistance 2; with help or training 1.5; administered by medical personnel 1) | 2 |
|  | Storage  conditions  (multiple choice)  (4 points) | Storage at room temperature | 3 |
|  |  | Storage in the shade | 2 |
|  |  | Refrigerated/frozen storage | 1 |
|  | Drug shelf life (2 points) | No need for shade/light protection | 1 |
|  |  | >60 months | 2 |
|  |  | ≥36 months, <60 months | 1.5 |
|  |  | ≥24 months, <36 months | 1 |
|  |  | ≥12 person-months, <24 months | 0.5 |
|  |  | <12 months | 0.25 |
| **Efficacy (27 points)** | Indications (5 points) | Clinically necessary, preferred | 5 |
|  |  | Clinical need, second choice | 3 |
|  |  | More medicines available | 1 |
|  | Guide recommendation (12 points) | Diagnosis and treatment norms/clinical pathways, consensus issued by national health administrative agencies/management methods, etc., guideline level I recommendation (Level A evidence 12; Level B evidence 11; Level C evidence, and others 10) | 12 |
|  |  | Guidelines Level II and below (Level A Evidence 9; Level B Evidence 8; Level C Evidence and Others 7) | 9 |
|  |  | Expert Consensus Recommendations (the consensus published by the society organizations based on systematic evaluation 6; the consensus published by the society organization others 4) | 6 |
|  |  | Systematic evaluation/Meta-analysis (large sample, high-quality systematic evaluation/Meta-analysis 3; small sample, low-quality systematic evaluation/  Meta-analysis 2; systematic evaluation/Meta-analysis of non-RCT studies 1). | 3 |
|  | Clinical efficacy (10 points) | The primary efficacy endpoint indicators | 6 |
|  |  | The secondary efficacy endpoint indicators | 4 |
| **Safety (25 points)** | Adverse reactions (multiple choices) (8) | Moderate adverse reactions: |  |
|  |  | Incidence <1% | 3 |
|  |  | Incidence 1% to <10% | 2 |
|  |  | Incidence ≥10% | 1 |
|  |  | ADR occurrence data not available | 0 |
|  |  | Severe adverse reactions: |  |
|  |  | Incidence < 0.01% | 5 |
|  |  | Incidence 0.01%~<0.1% | 4 |
|  |  | Incidence 0.1%~<1% | 3 |
|  |  | Incidence 1% to <10% | 2 |
|  |  | Incidence ≥10% | 1 |
|  |  | ADR occurrence data not available | 0 |
|  | Special groups  (multiple choice) (11 points) | Available for children (both 2; 1.9 for 3 months+; 1.8 for 6 months+; 1.7 for 9 months+; 1.6 for ages 1+; 1.5 for ages 2+; 1.4 for ages 3+; 1.3 for ages 4+; 1.2  for ages 5+; 1.1 for ages 6+; 1.0 for ages 7+; 0.9 for ages 8+; 0.8 for ages 9+ 0.7 for ages 10+; 0.6 for ages 11+; 0.5 for ages 12+. | 2 |
|  |  | The elderly (available 1; use with caution 0.5) | 1 |
|  |  | Pregnant women (early pregnancy 1; during the first trimester 0.8; during the second trimester 0.5). | 1 |
|  |  | Lactating women (available 1; use with caution 0.5) | 1 |
|  |  | Hepatic dysfunction (severe available 3, moderate  available 2. Lightly available 1) | 3 |
|  |  | Renal dysfunction (severe available 3, moderate  available 2. Lightly available 1) | 3 |
|  | Drug interaction (3 points) | No dosage adjustment is required | 3 |
|  |  | Dosage adjustment required | 2 |
|  |  | Prohibited to use at the same time | 1 |
|  | Other (multiple  choice) (3 points) | Reversibility of adverse reactions | 1 |
|  |  | No teratogenicity or carcinogenicity | 1 |
|  |  | No special medication warnings | 1 |
| **Economy (10 points)** | Same Generic Name Drug (3 points) | Daily treatment cost of the lowest-priced drug is 3 points; Evaluation drug score = Lowest daily treatment cost /average daily treatment cost of the evaluated drug × 3 | 3 |
|  | Alternative drugs with the main indications (7 points) | Daily treatment cost of the lowest-priced drug is 7 points; Evaluation drug score = Lowest daily treatment cost / average daily treatment cost of the evaluated drug × 7 | 7 |
| **Other attributes (10 points)** | National Health Insurance (3 points) | National medical insurance category A, no payment limitations | 3 |
|  |  | National medical insurance category A with payment limitations | 2.5 |
|  |  | National medical insurance category B, no payment limitations | 2 |
|  |  | National medical insurance category B with payment limitations | 1.5 |
|  |  | Not on the national health insurance catalog | 1 |
|  | National Essential Drugs (3 points) | National essential drugs without Δ requirement | 3 |
|  |  | National essential drugs with Δ Requirements | 2 |
|  |  | Not on the National Essential Drugs List | 1 |
|  | National Centralized Drug Procurement (1 point) | Selected drugs for centralized national procurement | 1 |
|  | Originator/Reference/Consistency Evaluation (1 point) | Drug of origin/reference drug | 1 |
|  |  | Generic drugs through consistency evaluation | 0.5 |
|  | Manufacturer ranking (1 point) | The world’s top 50 pharmaceutical manufacturers in terms of sales volume (1 for top 1-10; 0.8 for top 11- 20; 0.6 for top 21-30; 0.4 for top 31-40; 0.2 for top 41-50) / Top 100 Pharmaceutical Industry published by Ministry of Industry and Information Technology (1for top 1-20; 0.8 for top 21- 40; 0.6 for top 41-60; 0.4 for top 61-80; 0.2 for top 81-100). | 1 |
|  | Global Usage (1 point) | Available in China, the United States, Europe, Japan | 1 |
|  |  | Domestic and international sales | 0.5 |

**Supplementary Table 2 Search strategies for PubMed**

| **English Search terms** | |
| --- | --- |
| 1 | "Parkinson Disease"[MeSH] OR "idiopathic parkinson s disease"[Title/Abstractt] OR "parkinson disease idiopathic"[Title/Abstract] OR "parkinson disease idiopathic"[Title/Abstract] OR "parkinson disease"[Title/Abstract] OR "idiopathic parkinson disease"[Title/Abstract] OR "primary parkinsonism"[Title/Abstract] OR "parkinsonism primary"[Title/Abstract] |
| 2 | "Dopamine Agonists"[Mesh] OR "DA"[Title/Abstract] OR "non-ergot dopamine-receptor agonists"[Title/Abstract] OR "DAs"[Title/Abstract] OR "rotigotine" [Supplementary Concept] OR "rotigotine"[Title/Abstract] OR "ropinirole"[Supplementary Concept] OR "ropinirole"[Title/Abstract] OR "pramipexole"[Supplementary Concept] OR "pramipexole"[Title/Abstract] OR "piribedil"[Supplementary Concept] OR "piribedil"[Title/Abstract] |
| 3 | (("randomized controlled trial" [Publication Type] OR "controlled clinical trial" [MeSH Terms] OR "randomized controlled trials" [All Fields]) OR ("meta-analysis"[Publication Type] OR "meta-analysis as topic" [MeSH Terms] OR "meta-analysis"[All Fields]) |
| 4 | 1 AND 2 AND 3 |
